# Development and validation of an integrated wearable system for continuous cardiorespiratory monitoring using photoplethysmography and optical respiratory detection

**DOI:** 10.1007/s11517-026-03677-y

**Published:** 2026-09-25

**Authors:** Jesús David Ramírez, Nathalia Toro, Juan Manuel Campo, Guido Gómez-Peña, Julián Antonio Villamarín-Muñoz, Jaime A. Mosquera-Sánchez

**Affiliations:** 1 Program of Biomedical Engineering, Antonio Nariño University, Popayán, Colombia; 2 Doctoral Program in Applied Sciences, Antonio Nariño University, Bogotá DC, Colombia; 3 Department of Mechanical Engineering, São Carlos School of Engineering, University of São Paulo (USP), São Carlos, Brazil

**Keywords:** Photoplethysmography, Wearable devices, Cardiorespiratory monitoring, Heart rate variability, Mobile health

## Abstract

**Abstract:**

Cardiovascular diseases remain the leading cause of mortality worldwide, with ischaemic heart disease the leading contributor and acute myocardial infarction accounting for the majority of ischaemic heart disease deaths. This study presents the development and technical validation of a low-cost, integrated wearable system for multi-parameter cardiorespiratory monitoring designed to support heart-attack detection algorithms. The system comprises two devices: a wristband incorporating a MAX30105 photoplethysmography sensor for cardiac pulse-wave and SpO$_2$ acquisition, and a chest band featuring novel optical respiratory detection and an MPU6050 inertial measurement unit for respiratory rate and movement monitoring. Both devices use ESP32-C3 microcontrollers with Bluetooth Low Energy connectivity to a custom mobile application enabling real-time visualization and cloud-based storage. Validation was conducted against clinical-grade reference equipment (ADInstruments physiograph and EDAN iM8 monitor) in 43 healthy volunteers, yielding 50 acquisitions: 30 at rest and 20 post-physical activity. This is a technical feasibility study in healthy young volunteers; validation in clinical populations remains future work. Heart rate showed the closest agreement with the reference (percentage error ±1.94%, *r=0.99* post-activity; limits of agreement *-6.21* to 4.21 BPM), followed by respiratory rate (±3.47%, *r=0.99*). SpO$_2$ error remained within tolerance (1.64% at rest, 2.98% post-activity) but showed weak correlation with the reference owing to range restriction in a healthy cohort, and is not yet suitable for applications requiring absolute saturation values. This study demonstrates that a low-cost, multi-sensor wearable system can achieve accuracy meeting the prespecified feasibility criterion (*<± *5% error) in a healthy young cohort, establishing technical feasibility as a prerequisite to validation in the cardiovascular patient populations for which the system is ultimately intended.

**Graphical abstract:**

**Figure:**
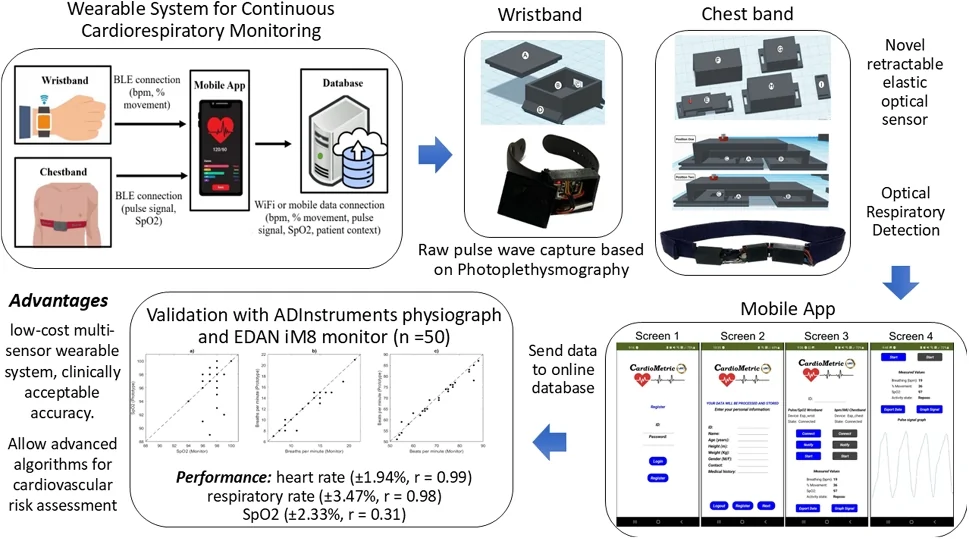

**Supplementary Information:**

The online version contains supplementary material available at https://doi.org/10.1007/s11517-026-03677-y.

## Introduction

Cardiovascular diseases (CVD) are the leading cause of death worldwide. According to the World Health Organization, an estimated 19.8 million people died from CVD in 2022, representing approximately 32% of all global deaths; 85% of these deaths were due to acute myocardial infarction (AMI) and stroke [1].

This condition occurs when blood flow and oxygen fail to reach the cardiac muscle, causing dysfunction and, in many cases, death. However, heart attacks can be prevented and treated with high survival probabilities if patients receive early medical attention or if cardiovascular risk can be estimated in advance [2]. Therefore, intervention time is crucial for survival, making prevention and early detection key research areas [3].

High mortality rates from heart attacks are partly due to healthcare system management challenges. Although symptomatology is the most evident way to identify heart attacks, it is not always clear. Common symptoms include intense chest pain, but more subtle signs such as difficulty breathing, dizziness, tachycardia, and sweating may also occur. Upon identifying symptoms, physicians follow a standard protocol for acute coronary syndrome [4], performing an electrocardiogram (ECG) and, if ST-segment changes are detected, requesting blood troponin tests. However, if no ECG changes appear, an echocardiogram is performed. Some patients do not return to hospitals after discharge, and waiting time for hospital transfer can limit effective treatment [3].

To mitigate high mortality rates, techniques with wearable and implantable devices have been developed for early diagnosis and remote monitoring [5]. Blood troponin detection is the most reliable method for identifying heart attacks, but it is invasive and requires specialized laboratories, implying high costs and prolonged waiting times [6]. Implantable biosensors exist but are uncommon and require costly surgeries [5]. Other techniques, such as phonocardiography using heart sounds to detect pathologies, have been explored. Puspasari et al. [7] propose a machine learning algorithm classifying patients into three groups based on signals recorded with a digital stethoscope. However, this technique identifies heart attacks that have already occurred, not ongoing ones, and requires expensive, non-portable equipment. Sathya and Magesh [8] use retinal images and artificial intelligence algorithms to assess heart-attack risk, but this method demands specialized equipment and patient preparation.

Devices using ECG have gained popularity due to their portability, low cost, and non-invasiveness. These devices, integrated with artificial intelligence, have proven effective in detecting heart attacks [9]. However, electrodes attached to the body pose inconveniences such as discomfort, skin irritation, and periodic replacement needs due to sweat or body hair [10]. Additionally, signal quality can be compromised by artifacts such as movement or electromagnetic interference [11], and correct electrode placement is crucial to avoid erroneous diagnoses [12]. Even with good quality signals, ECG does not detect all heart attacks, as significant ST-segment changes do not always occur, reducing diagnostic capacity to approximately 57% [13].

As an alternative to ECG, cardiac pulse-wave measurement through optical photoplethysmography (PPG) sensors has been proposed due to greater ease of use and lower noise [14]. Through this technique, heart rate and its variability (HRV) are obtained, useful for detecting arrhythmias, yet insufficient for identifying heart attacks [15]. Several works, such as those by Chong [16], Jianjiang et al. [17], and Gust et al. [18], propose using HRV to detect arrhythmias but not heart attacks. Recent studies by Shahnawaz and Dawood [19] and Kumar et al. [20] employ algorithms based on HRV and computational intelligence to classify patients according to cardiovascular status, yet their models are not focused on early detection and lack devices for real-time physiological data acquisition, using ECG signal databases available in the cloud.

Devices such as the Apple Watch [21], Garmin Smartwatch [22], and Oura Ring [23] have integrated health-monitoring functions, including heart rate, oxygen saturation (SpO$_2$), respiratory rate, temperature, and ECG. However, these devices are not designed for heart-attack detection or cardiovascular risk prediction. Although some provide HRV calculations, they do not offer raw photoplethysmography signals, which are useful for developing more accurate algorithms. Devices such as Polar OH1 [24] and EmbracePlus [25] provide raw photoplethysmography signals but lack specific focus on heart-attack prediction or detection.

We hypothesize that a low-cost, multi-sensor wearable system can achieve clinically acceptable accuracy (*<± *5% error) for cardiorespiratory monitoring when validated against medical-grade reference equipment. The specific objectives are: (1) to develop an integrated wearable system incorporating photoplethysmography, novel optical respiratory detection, and inertial measurement capabilities; (2) to validate system accuracy against clinical-grade reference equipment under rest and post-physical-activity conditions; (3) to establish operational ranges and performance metrics for all measured physiological parameters; (4) to demonstrate technical feasibility as a platform for advanced algorithms in cardiovascular risk assessment. Accordingly, the present work is a technical feasibility study: it establishes measurement accuracy in a controlled setting with healthy volunteers, which is the necessary first stage before validation in the elderly and cardiovascular patient populations that constitute the system’s target users.

This paper presents the development and validation of a wearable system based on low-cost, energy-efficient sensors for capturing cardiorespiratory variables. The system pairs photoplethysmographic and respiratory sensing with a custom mobile application to acquire pulse waveform, respiratory signal, oxygen saturation, movement status, and patient context. Its distinguishing feature is the retention of complete raw pulse and respiration waveforms rather than derived summary metrics alone. This enables offline extraction of statistical, temporal, spectral, and non-linear features that, combined with patient context, can serve as inputs to machine-learning models for cardiovascular risk assessment. Communication between the wearable devices and the mobile application uses Bluetooth Low Energy, while cloud upload proceeds over Wi-Fi or cellular networks. The devices can also connect autonomously over Wi-Fi and upload without an intermediary smartphone—an advantage for older adults and patients with limited mobility. Validation against clinical-grade reference equipment, presented in Section 3, establishes the measurement accuracy of the platform in a healthy cohort.

## Materials and methods

### Methodological design

For the development of this project, a Top-Down methodology was employed for the device design by starting from a global perspective and progressively breaking it down into specific and detailed components. The device validation was carried out using a quantitative and comparative methodology based on the correlation between measurements from the developed device and real values provided by validation standards, namely the ADInstruments physiograph and EDAN iM8 vital-signs monitor.

### System architecture

**Fig. 1:**
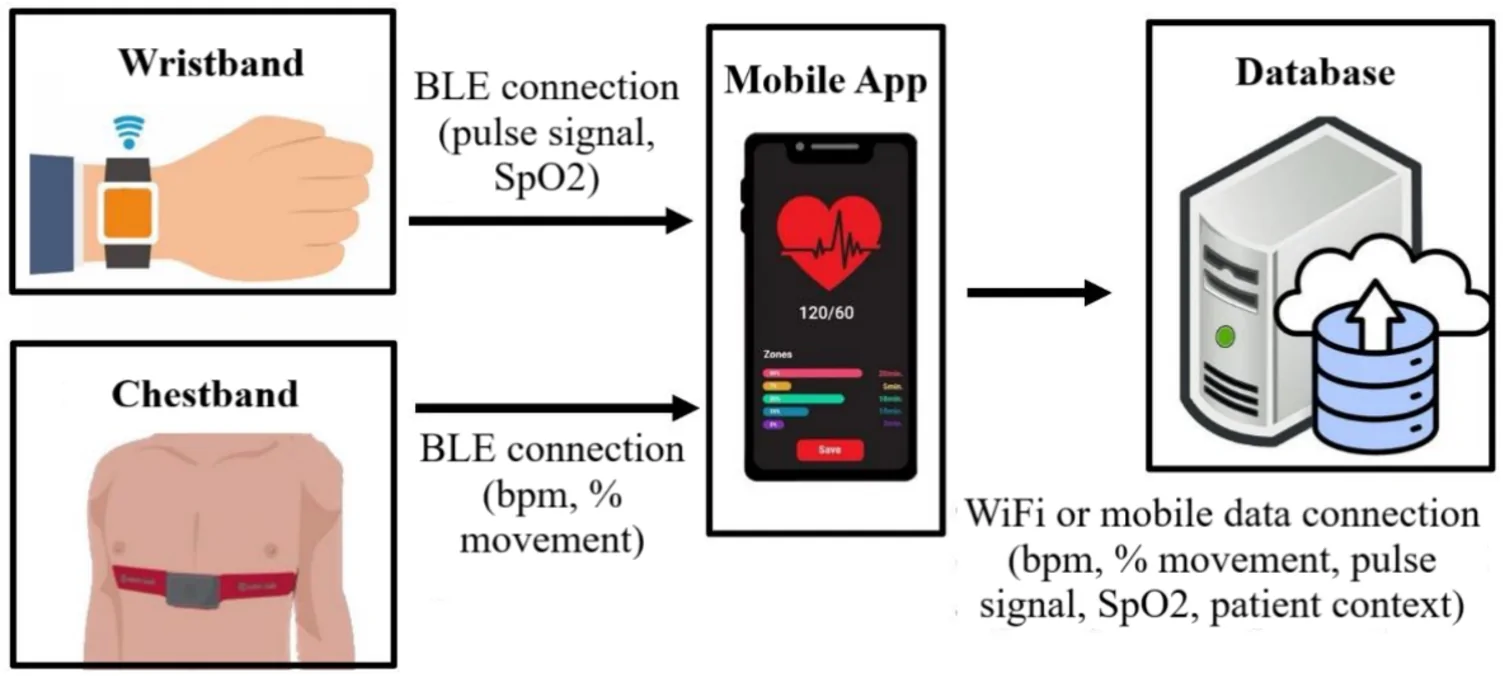
Block diagram of the system and dataflow

The developed system was structured into four integrated modules, as shown in Fig. 1. Module 1 consisted of a wrist band for SpO$_2$ and pulse-signal capture. Module 2 was a chest band for recording respiratory rate and patient movement status. Both devices transmitted data to Module 3 (mobile application) via Bluetooth Low Energy (BLE), where variables were visualized and patient context was entered. Finally, Module 4 consisted of a cloud database where all information was stored via Wi-Fi or mobile-data connection.

### Module 1: Wrist band device

#### Hardware and components

The wristband device relies on a MAX30105 sensor as the main acquisition component. This reflectance-mode sensor employs red, green, and infrared LEDs for heart-rate monitoring and pulse oximetry. An ESP32-C3 SuperMini microcontroller was implemented as the processing unit, a development board based on the ESP32-C3 chip with a 32-bit RISC-V single-core processor operating at up to 160 MHz, with 400 KB of SRAM and 4 MB of flash memory, compatible with Wi-Fi 802.11 b/g/n and Bluetooth 5.0 BLE. The selection of these components was based on their compact size, enabling an ergonomic device design.

**Fig. 2:**
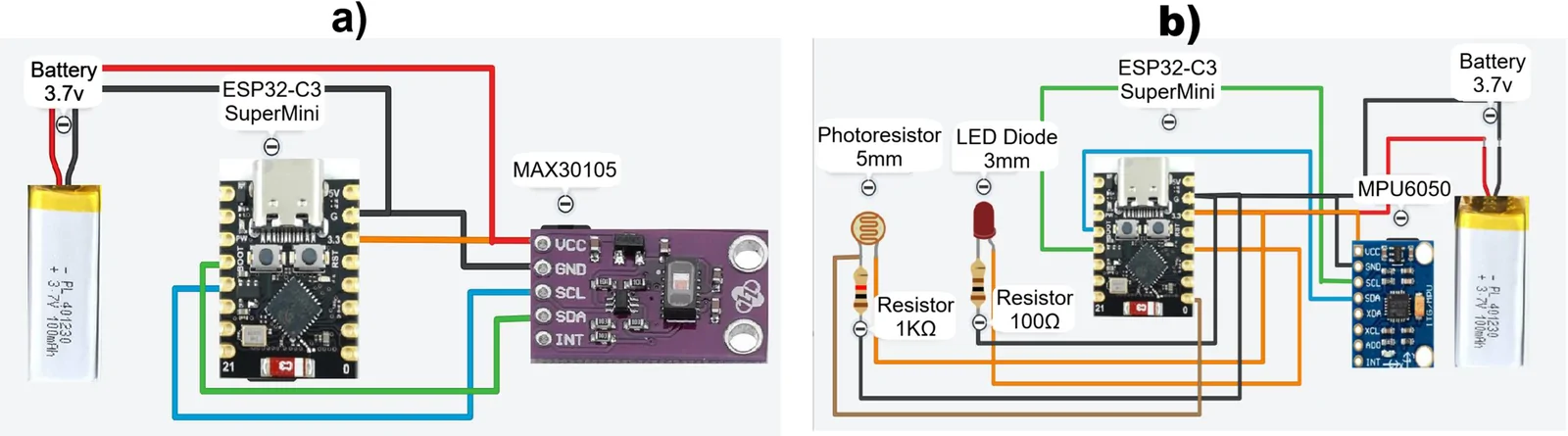
(**a**) Electrical circuit of Module 1 (wristband). (**b**) Electrical circuit of Module 2 (chestband)

**Fig. 3:**
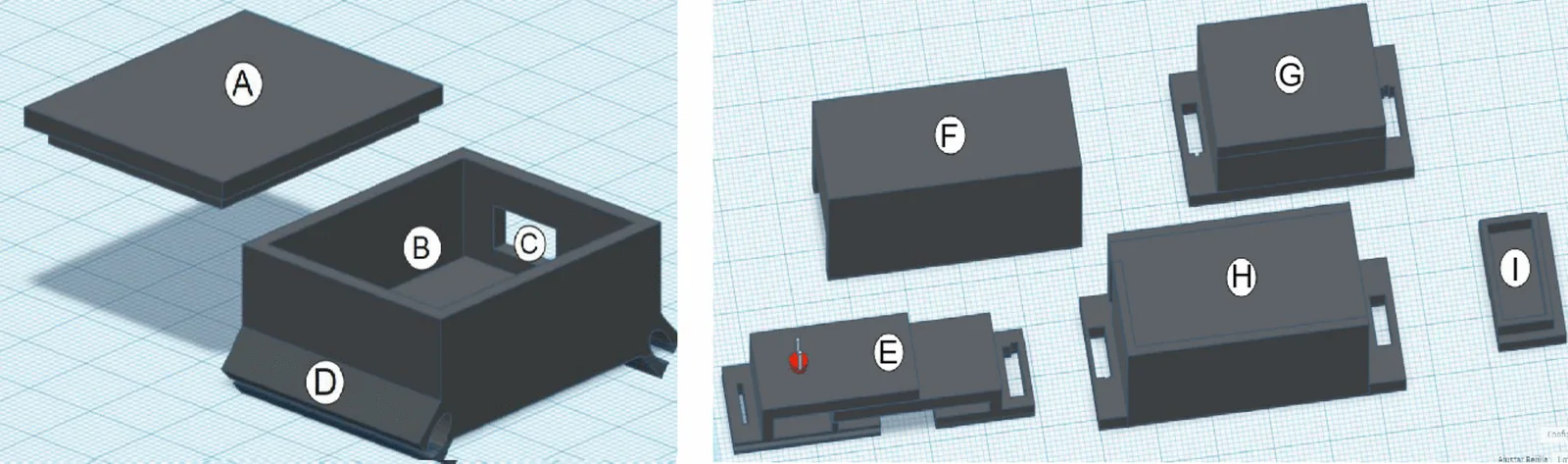
3D design of the wristband: (**A**) upper cover; (**B**) cavity for the circuit; (**C**) USB type-C input; (**D**) watch-strap slot. 3D design of the chestband: (**E**) retractable system; (**F**) retractable system covering; (**G**) controller compartment; (**H**) battery compartment; (**I**) battery cover

The electrical circuit integrated the MAX30105 sensor connected via I^2^C interface to the ESP32-C3 microcontroller, along with a rechargeable battery and watch straps, as shown in Fig. 2a. The 3D design consisted of two main pieces: an upper cover and a cavity for the electrical circuit with slots for watch-strap integration, as illustrated in Fig. 3.

The sensor was configured with all three LEDs active (red, infrared, and green; ledMode*= 3*), an LED pulse amplitude of 200 (of a 0–255 range, corresponding to a drive current of approximately 40 mA), a pulse width of 411 *μ *s (18-bit ADC resolution), an ADC full-scale range of 16,384 nA, an internal sampling rate of 100 Hz, and 8-sample averaging. A firmware delay fixed the effective output rate delivered to the pulse buffer at a measured 10 Hz. These settings were determined empirically by inspecting the resulting pulse-wave morphology while varying the LED intensity, and were selected to optimise pulse-wave quality for heart-rate-variability feature extraction rather than to match the manufacturer’s default SpO$_2$ configuration; the high LED drive current and the retention of the green LED reflect this choice, whose consequences for SpO$_2$ accuracy are examined in Section 4.1.1.

#### Processing algorithm

**Fig. 4:**
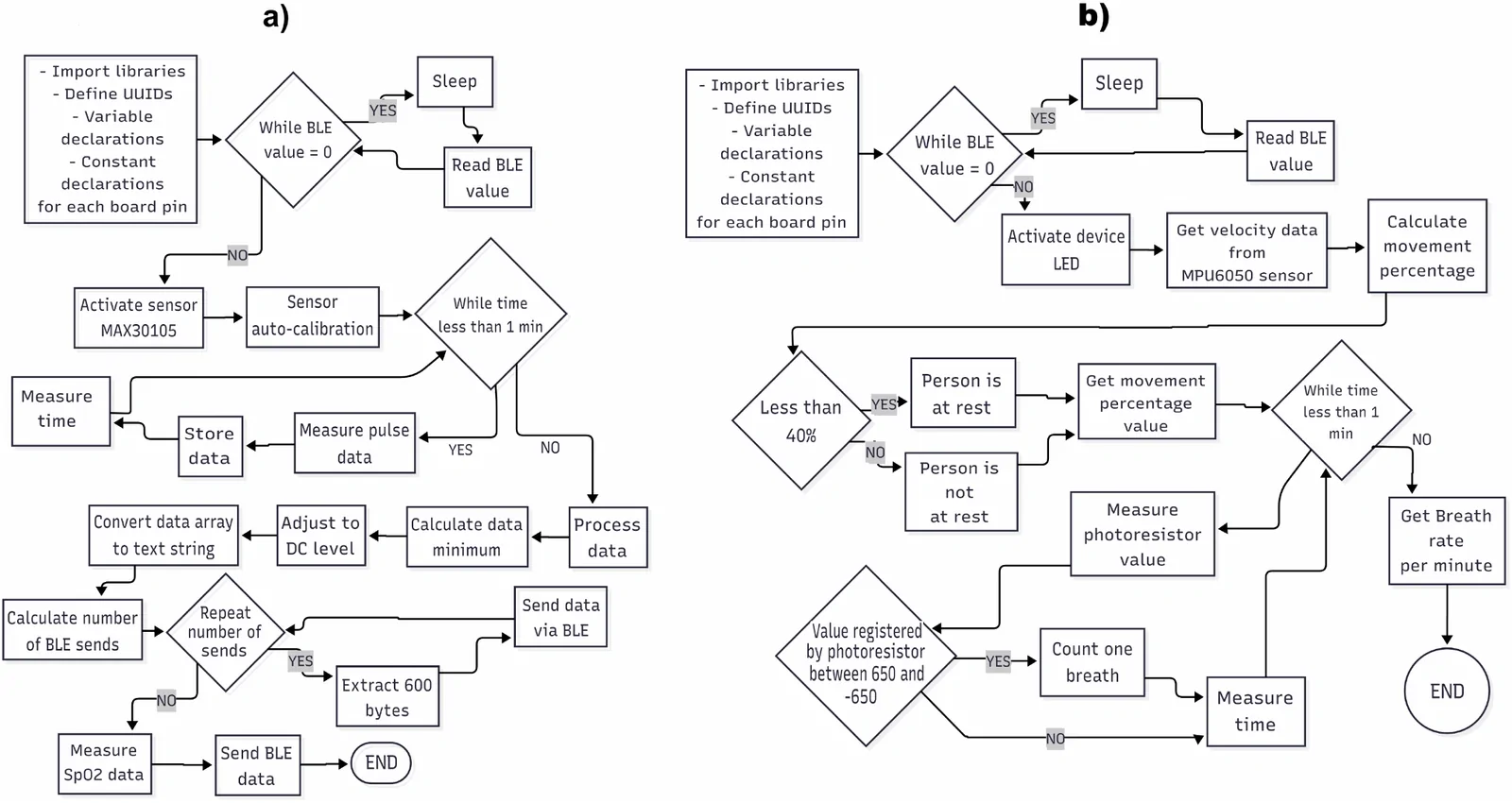
(**a**) Block diagram of the wristband device algorithm. (**b**) Block diagram of the chest band device algorithm

Figure 4a shows the wristband device algorithm, which was devised in the following stages: MAX30105 sensor initialization and Bluetooth Low Energy (BLE) communication establishment, followed by continuous acquisition of photoplethysmographic signals through the sensor’s LEDs for one minute, with all sampled values stored in a data array. Once data acquisition is complete, the array is processed to compute the signal minimum, which is subtracted from the entire signal in order to remove the DC offset and retain only the relevant photoplethysmographic components. This baseline correction also reduces the numerical range of the values, optimizing the data payload for transmission. The processed numerical array is then serialized into a comma-separated string, which is segmented into 600-byte packets and transmitted to the mobile application via BLE every 4 seconds. After the complete data string has been transmitted, SpO$_2$ is calculated from the stored signals using the ratio of red to infrared AC/DC components, and the resulting value is sent to the mobile device over BLE using a custom GATT profile. Upon completion of the transmission sequence, the ESP32-C3 enters an idle state, awaiting a new activation command.

### Module 2: Chest band device

#### Hardware and components

The chest band device featured an MPU6050 inertial measurement unit as the main sensor, a 6-degree-of-freedom IMU combining a 3-axis accelerometer and 3-axis gyroscope, configured at a gyroscope full-scale range of $\pm 250~{}^{\circ }\text {/s}$ (sensitivity 131 LSB per ${}^{\circ }\text {/s}$) and read at 10 Hz. The Y-axis gyroscope was used for detecting user movements and calculating the patient’s movement percentage.

For respiratory-rate measurement, a retractable system was designed based on optical detection using a 3 mm red LED and photoresistor. The LED limiting-resistor calculation was performed using Ohm’s Law:

1 $$\begin{aligned} R(\Omega ) = \frac{V_{\text {supply}}(V) - V_{\text {LED drop}}(V)}{I_{\text {nominal LED}}(A)} = \frac{3.3V - 2.0V}{0.02A} = 65\Omega \end{aligned}$$

A 100 *Ω * resistor was implemented as the closest commercial value. The electrical circuit shown in Fig. 2b connected the photoresistor to the microcontroller’s ADC (12 bits), obtaining a digital variation from 215 to 4095 between contracted and stretched position, corresponding to an operating range of 3880 (digital value) with a resolution of 3 *μ *m.

#### Mechanical respiratory detection system

Figure 3 illustrates the band’s 3D design with six parts: retractable system with light-measurement circuit, retractable system covering, microcontroller compartment, battery compartment, battery cover, and fastening system using velcro and ribbon tape.

**Fig. 5:**
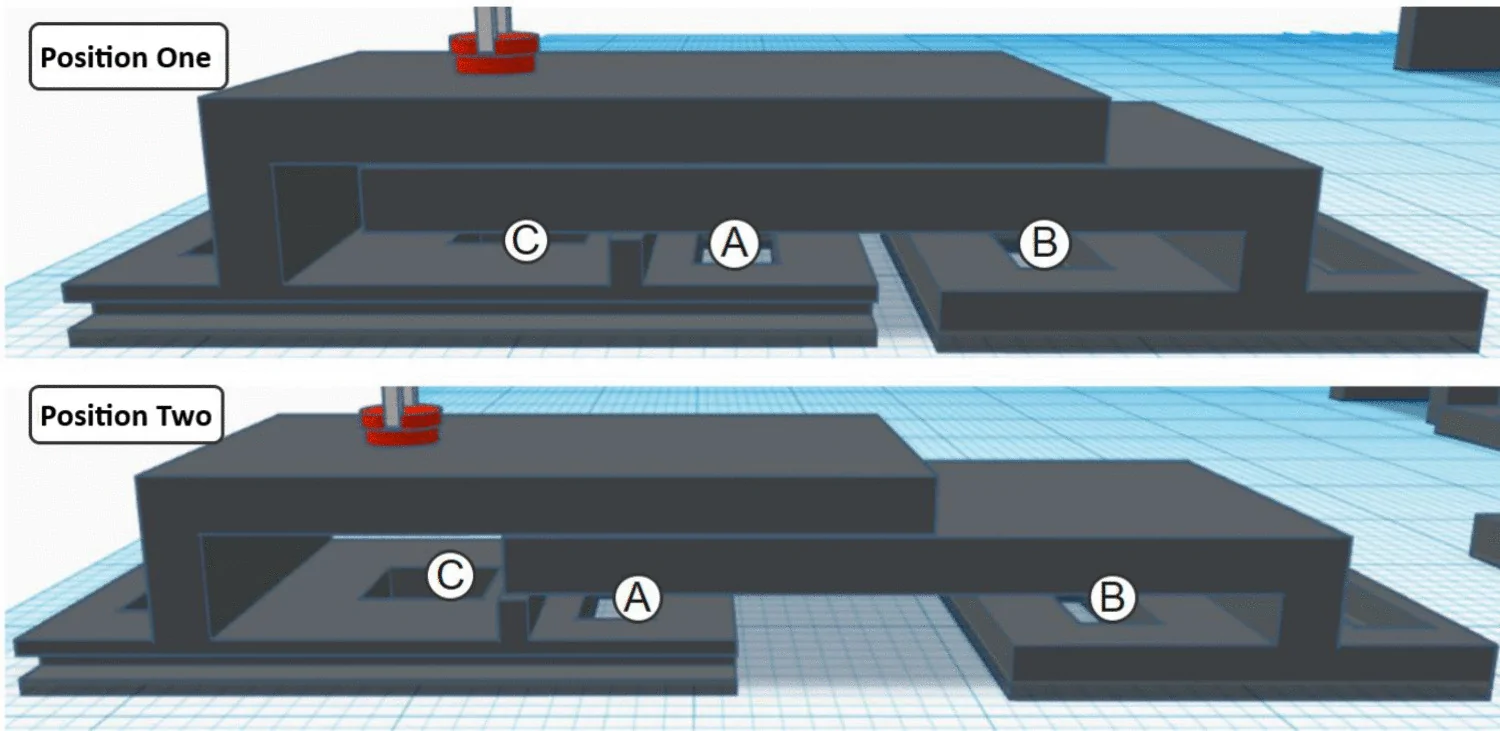
3D images of the retractable system. (**A**) left elastic band slot; (**B**) right elastic band slot; (**C**) photodetector slot

The retractable system (Fig. 5) operated through two positions: position one for rest and pre-inspiration with structure overlap preventing light passage; and position two for inspiration, with thoracic expansion allowing 0–12 mm displacement, thus generating light passage and respiration recording through light-detection event counting.

#### Processing algorithm

The chest band algorithm (Fig. 4b) initialises the MPU6050 and the optical detection circuit, then acquires both channels over a 60-second window at 10 Hz (*N = 600* samples).

Movement percentage was computed as the fraction of samples in which the absolute Y-axis angular rate exceeded a threshold of 650 raw units, equivalent to approximately $5~{}^{\circ }\text {/s}$ at the configured full-scale range:

2 $$\begin{aligned} M = \frac{100}{N}\sum _{n=1}^{N} \mathbb {1}\!\left( \left| \omega _y[n]\right| > 650\right) , \end{aligned}$$

where $\mathbb {1}(\cdot )$ denotes the indicator function. The participant was classified as active when $M \ge 40\%$ and at rest otherwise.

Respiration was counted on-device as the number of upward transitions of the photoresistor ADC value across a threshold of 500 counts within the same window, each transition corresponding to one complete inspiration. This threshold lies near the lower end of the operating range of the optical assembly—215 counts with the sliding structures fully overlapped and 4095 counts fully separated—so that counting is triggered at the onset of light passage rather than at full thoracic expansion. Respiratory rate, movement percentage, and activity status were then transmitted to the mobile application over Bluetooth Low Energy.

### Module 3: Mobile application

#### Software architecture

**Fig. 6:**
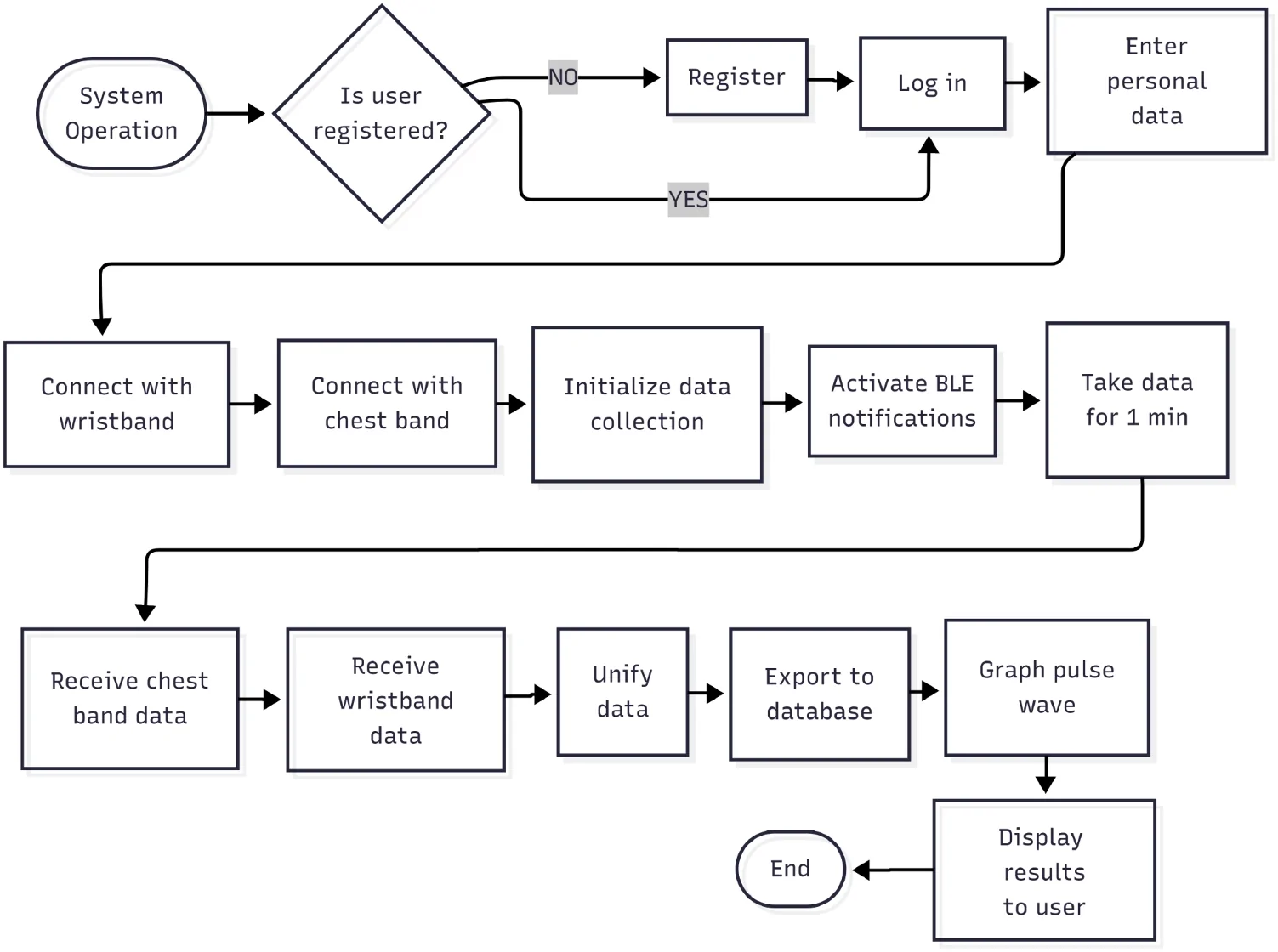
Block diagram of the application operating algorithm

The mobile application was developed in MIT App Inventor. The operational flow shown in Fig. 6 comprised unique patient registration through ID number and password, personal data entry, guardian contact information and clinical history, independent Bluetooth BLE connection with each device, real-time visualization of cardiorespiratory variables (RPM, movement percentage, activity status, pulse-wave graph), and data export to cloud database.

#### User interface

**Fig. 7:**
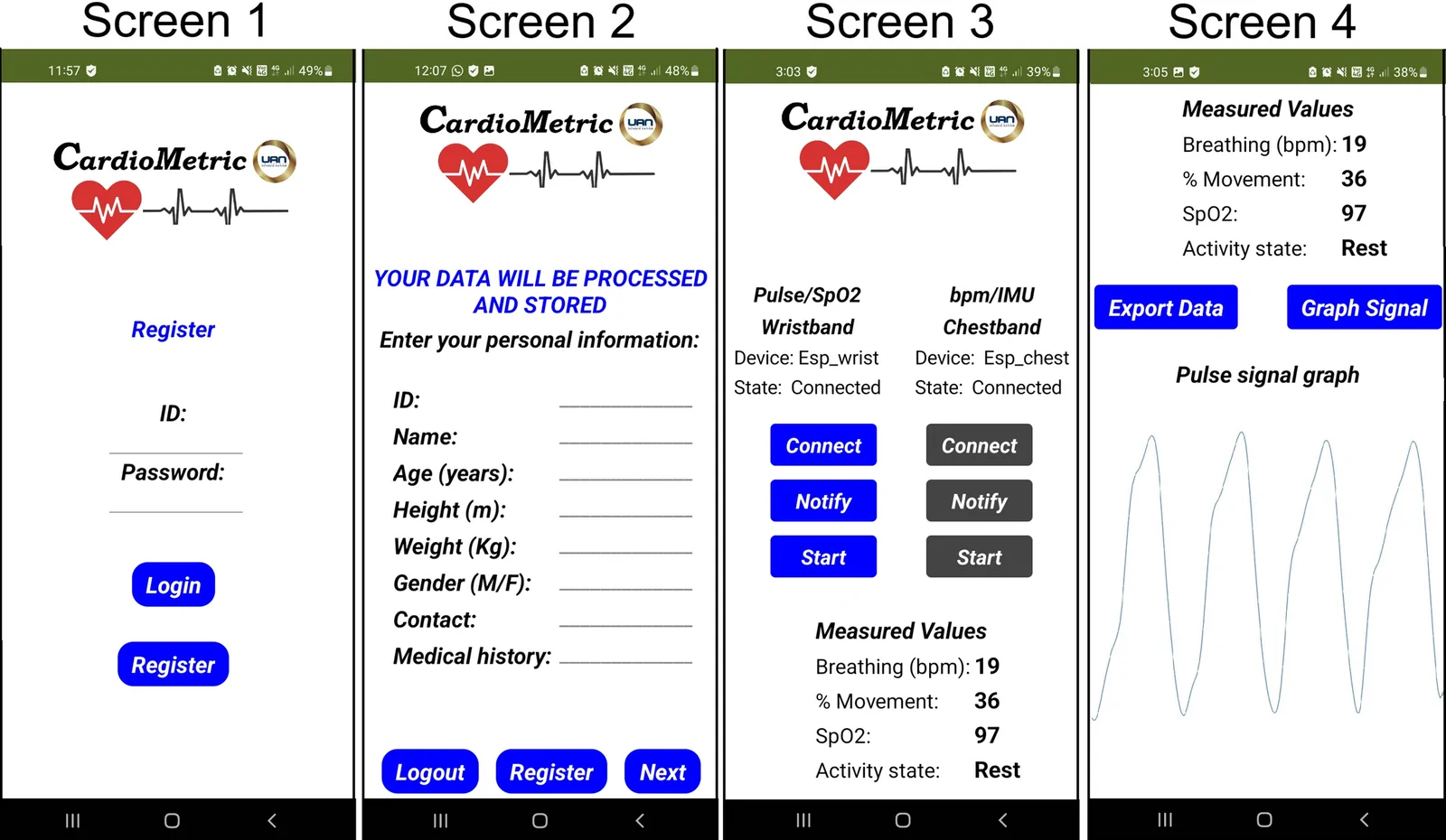
Images of the application’s graphical user interface (GUI). (Screen 1) user validation; (Screen 2) user personal data; (Screen 3) BLE sensors manager; (Screen 4) results visualization

The graphical user interface (GUI) was structured into three areas: upper area with application title, central area with specific content, and lower area with menu buttons. Screen 1 presents the registration form and login. Screen 2 allows user’s personal and medical information entry. Screen 3 integrates Bluetooth connection sections featuring pairing and data-reception buttons for both devices, band measurement visualization, pulse-wave graph, and database-export buttons (Fig. 7). Data processing was optimized by segmenting the pulse signal and transmitting it in 4-second intervals.

#### Test platform

The application was debugged and installed on a Samsung Galaxy A21s device with Android 12 operating system, verifying login functionality, visualization, data export, and BLE connectivity with prototypes.

### Module 4: Database

Data management was implemented on the 000webhost hosting platform using MySQL engine and administration through PHPMyAdmin. The database structure stored: patient identification, personal data, contact information, medical history, and temporal records of SpO$_2$, heart rate, pulse wave, respiratory rate, movement percentage, and activity status. Transmission from the mobile application was activated via the “Export Data” button on Screen 3, sending data through HTTP POST protocol.

### Pulse signal processing

Pulse-wave analysis was not performed on the wearable device. The wristband transmitted the raw photoplethysmographic (PPG) waveform to the mobile application, which relayed it to the cloud database; only SpO$_2$ was computed on-device, as described in Section 2.3.2.

Heart rate was derived offline in MATLAB R2024b (Signal Processing Toolbox) from the stored waveform, with the sampling frequency supplied explicitly as the measured effective acquisition rate of 10 Hz. The comma-separated signal string was parsed into a numeric vector and band-pass filtered to isolate the cardiac component: a fourth-order Butterworth band-pass filter with cut-off frequencies of 0.5 and 4.0 Hz—corresponding to a physiological range of 30 to 240 beats per minute—was designed by the bilinear transform and applied with forward–backward (zero-phase) filtering, giving an effective magnitude response of order eight without phase distortion. Systolic peaks were located on the filtered signal using an amplitude threshold set dynamically at the signal mean plus 0.3 times its population standard deviation, together with a minimum inter-peak separation derived from the 240 beats-per-minute ceiling. Heart rate was computed from the mean inter-peak interval; if fewer than two peaks were detected, a fallback estimate was obtained from the total peak count over the acquisition duration. The complete pseudocode is given in Appendix B, and identical parameters were applied to both the prototype signal and the ADInstruments piezoelectric reference signal, so that any residual bias in peak identification affects both equally.

The retention of the complete raw waveform, rather than of derived summary metrics alone, is a deliberate design decision and the principal architectural distinction of the proposed system. It places the choice of filtering, interpolation, and feature-extraction strategy with the analyst rather than fixing it in firmware, and permits the same acquisition to be re-analysed as processing methods evolve.

The acquisition rate is itself a configurable firmware parameter rather than an architectural constraint. The MAX30105 supports internal sampling rates from 50 to 3200 Hz with configurable sample averaging, and the effective rate delivered to the transmission buffer is governed by a firmware delay; the 10 Hz used for the present validation was selected to bound the transmitted payload, and higher rates are obtained by adjusting this delay together with the sensor’s averaging register. The governing trade-off is transmission time and energy consumption rather than hardware capability, since the serialised payload—and therefore the number of 4-second BLE segments required—scales linearly with the acquisition rate.

Within the configuration validated here, the acquisition envelope is defined by the 10 Hz rate together with the 0.5–4.0 Hz analysis band: the Nyquist criterion is satisfied for the retained band, so that resampling—by spline interpolation, for instance—reconstructs the band-limited waveform and refines peak localisation to sub-sample precision without introducing information absent from the acquisition. Inter-beat interval series obtained in this manner support statistical, temporal, and non-linear variability indices, together with spectral analysis of the interval series. Analyses that depend on pulse-waveform morphology above 5 Hz, such as second-derivative indices, would require the higher acquisition rate that the firmware already permits.

### Validation protocol

#### Reference equipment

Validation devices were the ADInstruments physiograph with respiratory-rate sensor and piezoelectric sensor for pulse signal, and the EDAN iM8 vital-signs monitor with SpO$_2$ sensor. These equipment were considered measurement standards for comparison with the developed devices.

#### Evaluation metrics

The device functionality was evaluated through three statistical parameters, namely Root Mean Square Error (RMSE), Accuracy (mean absolute percentage error), and Pearson Correlation Coefficient, calculated as follows:

3 $$\begin{aligned} \begin{aligned} \text {RMSE} = \sqrt{\frac{\sum (O_i - E_i)^2}{n}};&\quad E = \frac{|\text {Data}_{\text {prototype}} - \text {Data}_{\text {V.Device}}|}{\text {Data}_{\text {V.Device}}} \times 100;\\ \rho _{XY} =&\frac{\text {Cov}(X, Y)}{\sqrt{\text {Var}(X) \times \text {Var}(Y)}}, \end{aligned} \end{aligned}$$

where $O_i$ represents the real value, $E_i$ the predicted value, and *n* the number of elements. The coefficient interpretation followed the ranges established in Table 1.

In addition to these metrics, agreement between the developed devices and the reference standards was assessed by Bland–Altman analysis [27]. For each parameter, the difference between paired measurements was plotted against their mean; bias was estimated as the mean difference and 95% limits of agreement (LoA) as $\text {bias} \pm 1.96\,\text {SD}_{\text {diff}}$, with 95% confidence intervals computed for the bias and for each limit. Proportional bias was tested by regressing the differences on the means. Bland–Altman analysis is reported alongside correlation because the Pearson coefficient quantifies association rather than agreement and is attenuated under range restriction, and is therefore not a sufficient basis for method comparison.

A mean absolute percentage error below ±5% was adopted a priori as the feasibility criterion for this study, consistent with the hypothesis stated in Section 1. This threshold defines the study’s success criterion in a healthy cohort and is not presented as a regulatory or clinical acceptance limit.

**Table 1:** Interpretation of Pearson correlation coefficient

Range of $\rho _{XY}$ values	Interpretation
$0.00 \le |\rho _{XY}| < 0.10$	Null correlation
$0.10 \le |\rho _{XY}| < 0.30$	Weak correlation
$0.30 \le |\rho _{XY}| < 0.50$	Moderate correlation
$0.50 \le |\rho _{XY}| < 1.00$	Strong correlation

#### Participants and experimental protocol at rest and post-activity

**Duration:** 1 minute per test.

**Groups:** (1) rest, (2) post-physical activity.

**Procedure:** Patients were positioned in supine position with arm supported on a flat surface. The following were placed simultaneously: wristband device and SpO$_2$ sensor from EDAN iM8 monitor on the wrist; chest band device, respiratory-rate sensor, and piezoelectric sensor from ADInstruments on the thorax. For the post-activity group, patients ran approximately 100 meters prior to measurement. During acquisitions, patients remained motionless in supine position.

**Participants and sample size:** Forty-three healthy volunteers were recruited from Antonio Nariño University (age range 19–28 years; 20 male, 23 female). Thirty completed the rest protocol (15 male, 15 female) and twenty completed the post-activity protocol (8 male, 12 female); seven participants completed both, yielding 50 acquisitions from 43 individuals. Because the overlap is limited to seven participants, the two conditions are treated as largely independent samples, and no paired analysis between conditions is reported. The sample size was determined pragmatically on the basis of volunteer availability and is consistent with comparable wearable-validation studies [26].

**Sensitivity analysis:** Rather than computing observed post hoc power, which is a monotone transformation of the *p*-value and therefore uninformative, we report the sensitivity of each condition to pre-specified effect sizes. Using the Fisher *z* transformation with *α = 0.05* (two-tailed), the rest condition (*n = 30*) provides 81% power to detect a correlation of *ρ = 0.5* and above 99% power for *ρ = 0.8*; the post-activity condition (*n = 20*) provides 62% and above 99% respectively. The study is therefore well powered for the strong associations observed for heart rate and respiratory rate, and adequately powered to have detected a moderate association for SpO$_2$ had one been present, but it is underpowered for weak associations and for the sex-stratified comparison reported in Section 3.5.3.

**Repeated acquisitions:** Three acquisitions produced physiologically implausible values, traced to depletion of the wristband battery. The device was recharged and these acquisitions were repeated; the repeated measurements are the ones reported. The original acquisitions were discarded rather than averaged, and no other acquisition was repeated or excluded. This incident motivates the recommendation in Section 4.4 that subsequent versions implement battery-state monitoring and a signal-quality index, since a low-battery artefact is not distinguishable from a genuine desaturation on the basis of the reported value alone.

#### Inertial unit experimental protocol

**Duration:** 1 minute.

**Conditions:** Six sessions: (1) sitting without movement, (2) sitting with peripheral limb movement, (3) movement sequence (sitting 15s, walk 10 steps, sitting 5s, walk 10 steps, sitting until completing 1 minute), (4) walking, (5) climbing stairs, (6) running.

**Objective:** Evaluate IMU response according to status (rest/activity) and associated movement percentage.

## Results

### Device implementation

**Fig. 8:**
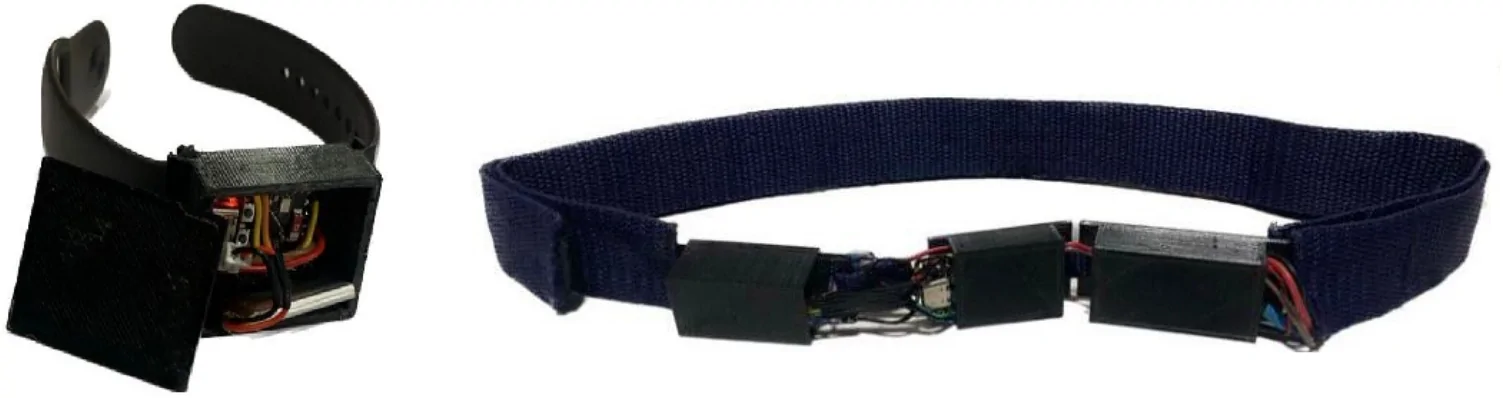
Images of the developed prototype, chest band on the right and wristband on the left

The final devices were built following established 3D designs. The wristband and chest band prototypes (Fig. 8) successfully integrated all components including sensors, microcontrollers, batteries, and fastening systems.

### Mobile application performance

The developed mobile application allowed successful registration of patient information and real-time visualization of cardiorespiratory variables. Cloud database transmission functioned successfully, with pulse-signal processing optimized through data segmentation with transmission in 4-second intervals. In the 50 acquisitions performed, no information loss was evident during transmission and storage.

### Rest test analysis

#### Physiological data distribution

**Fig. 9:**
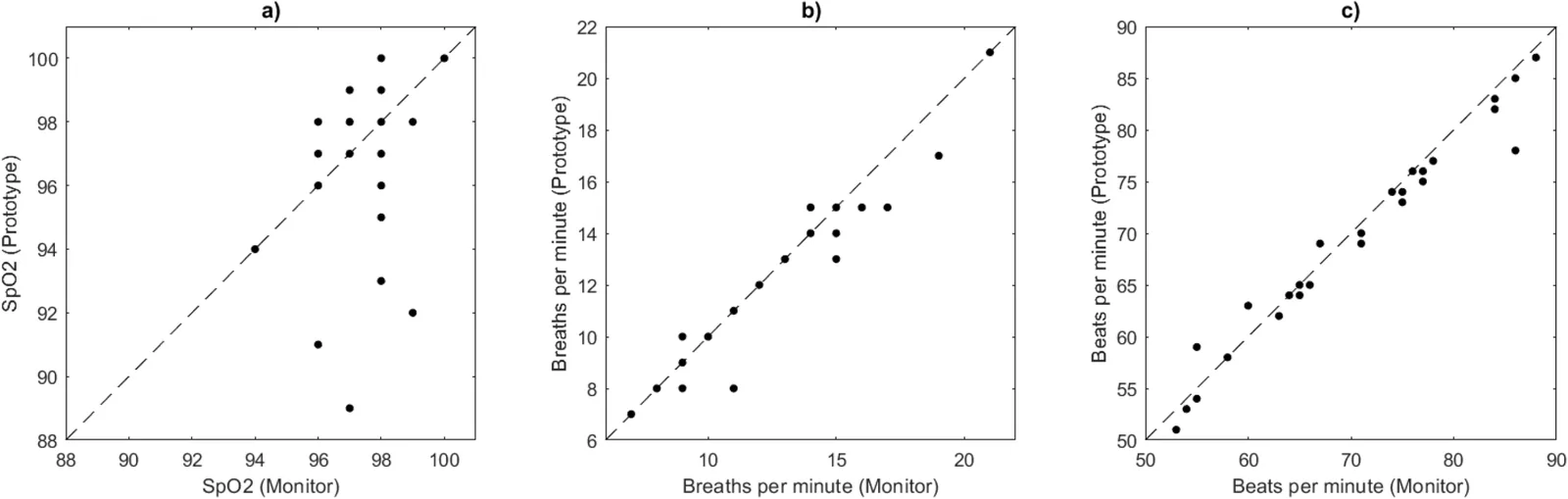
Performance comparisons between the proposed system and the reference equipment at rest. (**a**) SpO$_2$; (**b**) respiratory rate; (**c**) heart rate. Dots represent correlation between the proposed and the reference systems, while the dotted lines represent the validation-standard trend

In the rest condition (*n = 30*), reference SpO$_2$ ranged from 94% to 100% and prototype readings from 89% to 100% (Fig. 9a); respiratory rate from 7 to 21 RPM (Fig. 9b); and heart rate from 53 to 88 BPM (Fig. 9c). Scatter plots evidenced correspondence between developed-device measurements and validation standards.

#### Signal validation

**Fig. 10:**
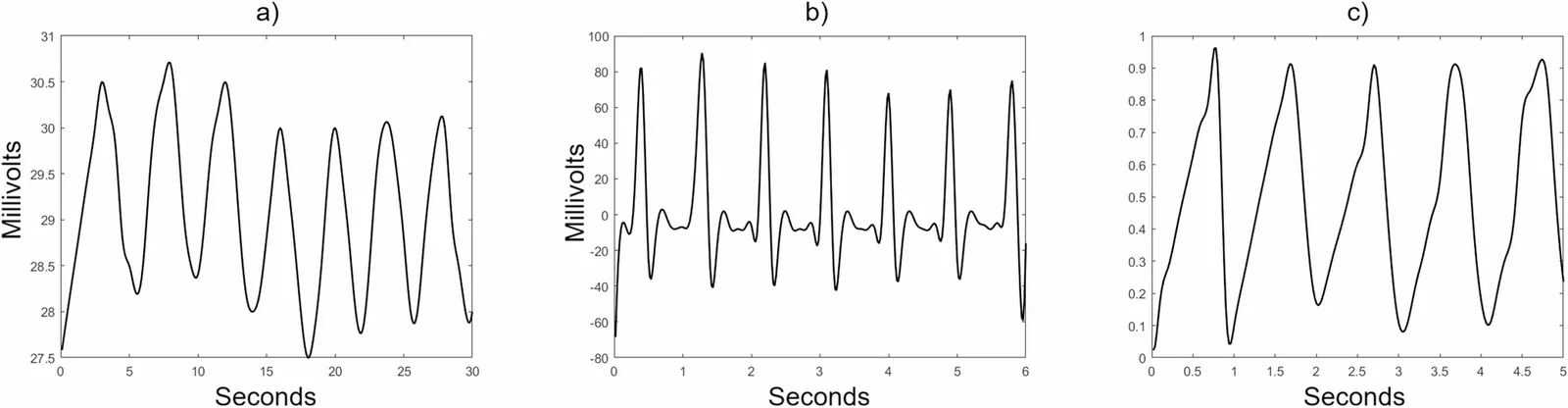
Raw signals of the ADInstruments device. (**a**) respiratory rate and (**b**) heart rate; compared with the peaks count strategy, illustrated in panel (**c**)

Respiratory-rate analysis was performed by counting peaks in the ADInstruments respiratory-sensor signal (Fig. 10a), comparing with chest-band device respiration counts. For heart rate, peak counting of the ADInstruments piezoelectric signal (Fig. 10b) and MAX30105 signal processed in MATLAB (Fig. 10c) was performed, using the pipeline described in Section 2.7. SpO$_2$ was validated by comparing the final value of the EDAN iM8 monitor with the wristband device measurement.

### Post-physical activity test analysis

#### Physiological data distribution

**Fig. 11:**
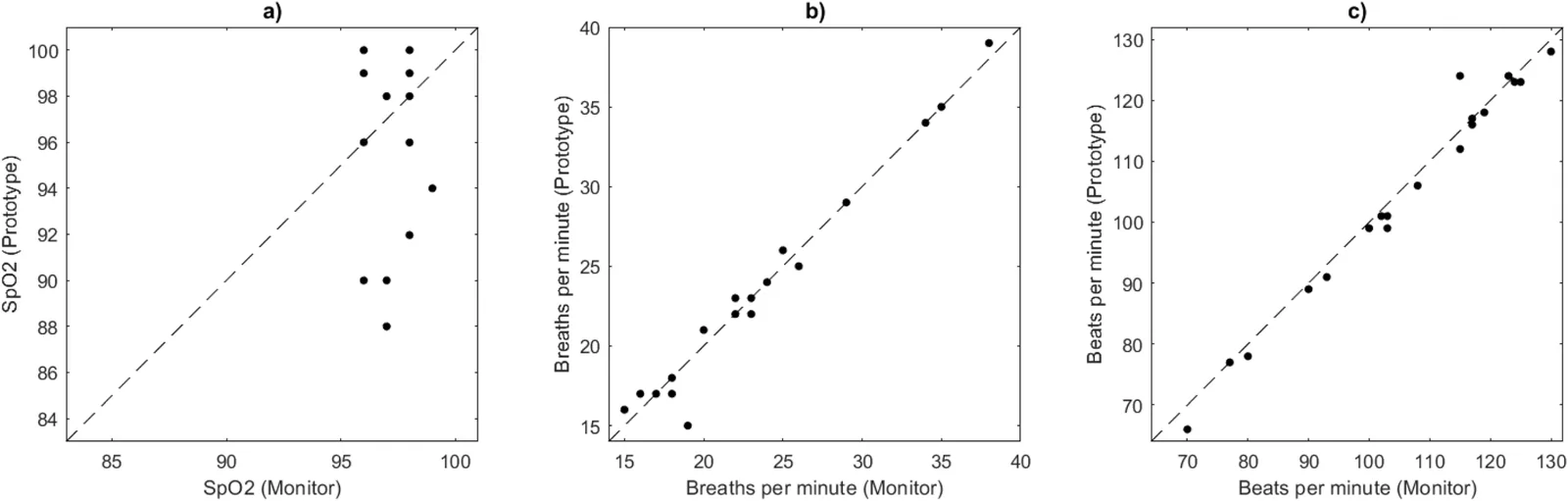
Performance comparisons between the prototype and the reference equipment after physical activity. (**a**) SpO$_2$; (**b**) respiratory rate; (**c**) heart rate. Dots represent correlation between the proposed and the reference systems, while the dotted lines represent the validation-standard trend

In the post-activity condition (*n = 20*), reference SpO$_2$ ranged from 96% to 99% and prototype readings from 88% to 100% (Fig. 11a); respiratory rate from 15 to 38 RPM (Fig. 11b); and heart rate from 70 to 130 BPM (Fig. 11c). Heart rate and respiratory rate were elevated relative to the rest condition, consistent with the expected cardiovascular response to exercise, whereas reference SpO$_2$ occupied an even narrower band than at rest—a point relevant to the interpretation of the correlation statistics (Section 4.1.1).

### Performance metrics

#### Global statistical results

Table 2 presents validation metrics for both experimental conditions. At rest, SpO$_2$ showed RMSE of 2.61, mean absolute percentage error of 1.64%, and a Pearson coefficient of 0.35 (*p = 0.058*, moderate). Respiratory rate presented RMSE of 1.03, error of 4.69%, and Pearson coefficient of 0.96 (*p < 0.001*, strong). Heart rate recorded RMSE of 2.08, error of 1.99%, and Pearson coefficient of 0.98 (*p < 0.001*, strong).

Post-physical activity, SpO$_2$ showed RMSE of 3.77, error of 2.98%, and a Pearson coefficient of 0.21 (*p = 0.379*, weak). Respiratory rate presented RMSE of 1.14, error of 3.47%, and Pearson coefficient of 0.99 (*p < 0.001*, strong). Heart rate recorded RMSE of 2.77, error of 1.94%, and Pearson coefficient of 0.99 (*p < 0.001*, strong).

**Table 2:** Validation metrics for all measured physiological parameters

Parameter	Condition	RMSE	MAPE (%)	Pearson *r*	*p*-value	Relation
SpO$_2$ (%)	Rest	2.61	1.64 ± 2.15	0.35	0.058	Moderate
	PA	3.77	2.98 ± 2.55	0.21	0.379	Weak
RR (RPM)	Rest	1.03	4.69 ± 6.74	0.96	<0.001***	Strong
	PA	1.14	3.47 ± 4.86	0.99	<0.001***	Strong
HR (BPM)	Rest	2.08	1.99 ± 2.11	0.98	<0.001***	Strong
	PA	2.77	1.94 ± 1.92	0.99	<0.001***	Strong

Rest condition: *n = 30* acquisitions; PA (post-activity): *n = 20* acquisitions. RMSE expressed in the units of each parameter. MAPE = mean absolute percentage error (mean ± SD). *r* = Pearson correlation coefficient; ***p < 0.001. Correlation strength classified per Table 1. Reference standards: ADInstruments physiograph (RR, HR); EDAN iM8 monitor (SpO$_2$). MAPE remained below the ±5% threshold adopted as the prespecified feasibility criterion of this study for all parameters under both conditions. Agreement (Bland–Altman) analysis for the same data is reported in Table 3

#### Agreement analysis

**Fig. 12:**
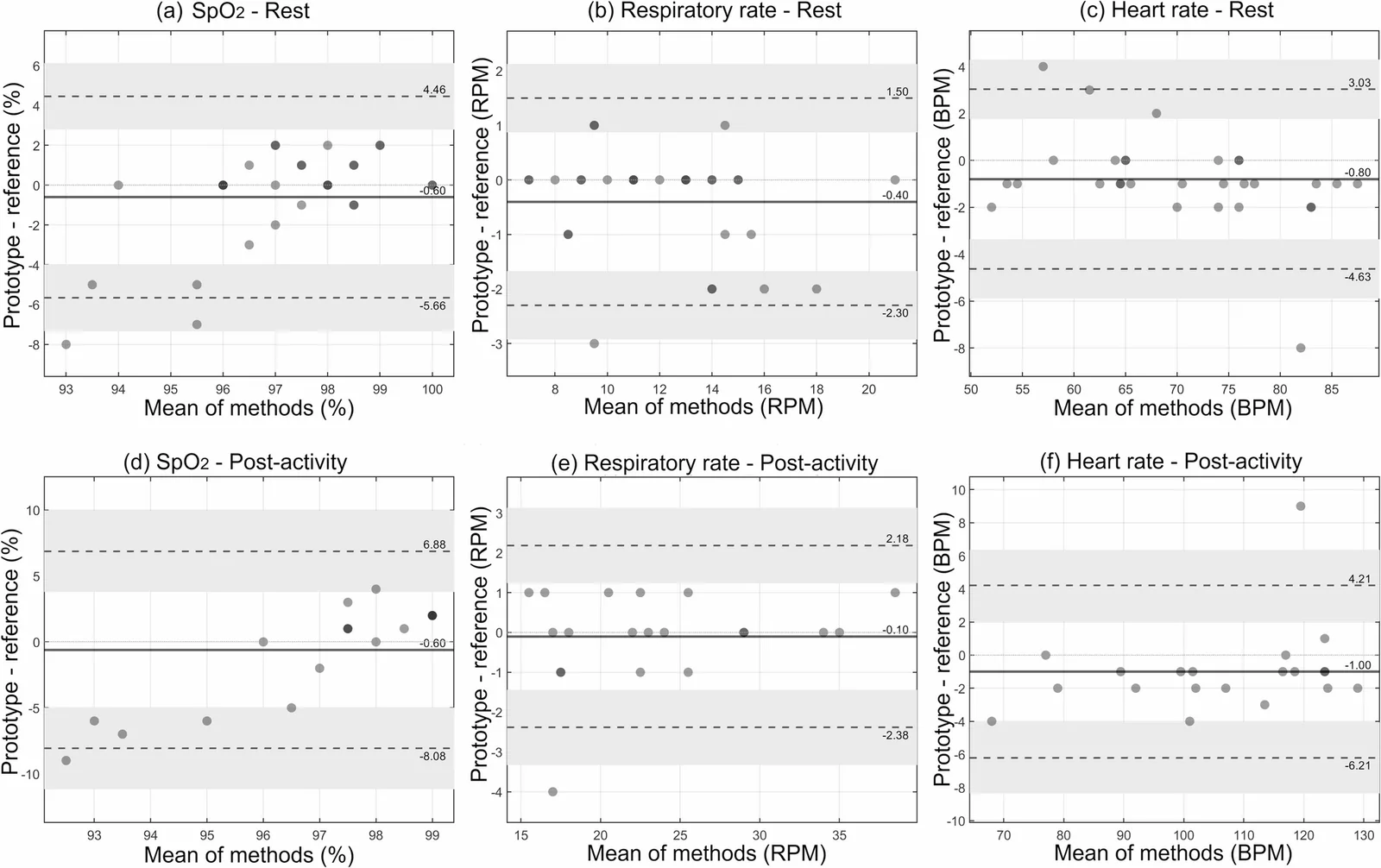
Bland–Altman plots comparing the proposed system with the reference equipment. Top row, rest condition (*n=30*); bottom row, post-activity condition (*n=20*). (**a**, **d**) SpO$_2$; (**b**, **e**) respiratory rate; (**c**, **f**) heart rate. Solid line: bias; dashed lines: 95% limits of agreement; shaded bands: 95% confidence intervals of the limits

Bland–Altman analysis (Fig. 12, Table 3) quantified agreement between the developed devices and the reference standards. All parameters showed a small negative bias, indicating slight systematic underestimation by the prototype.

At rest, respiratory rate showed a bias of *-0.40* RPM (95% CI: *-0.76* to *-0.04*) with 95% limits of agreement (LoA) of *-2.30* to 1.50 RPM, and heart rate a bias of *-0.80* BPM (95% CI: *-1.53* to *-0.07*; LoA: *-4.63* to 3.03 BPM). Both intervals are narrow relative to the physiological range of each variable. SpO$_2$ at rest showed a bias of *-0.60* percentage points (95% CI: *-1.56* to 0.36) with LoA of *-5.66* to 4.46.

Post-activity, respiratory rate (bias *-0.10* RPM; LoA: *-2.38* to 2.18) and heart rate (bias *-1.00* BPM; LoA: *-6.21* to 4.21) retained agreement comparable to the rest condition. SpO$_2$ showed the widest limits of agreement in the study (*-8.08* to 6.88; bias *-0.60*), with five of twenty acquisitions deviating by 5 percentage points or more and a maximum underestimation of 9 points.

Regression of differences on means indicated no significant proportional bias for respiratory rate under either condition or for heart rate post-activity (all *p > 0.19*); a weak proportional relationship was detected for heart rate at rest (slope *-0.076*, *p = 0.033*), of negligible magnitude across the observed range. The equivalent analysis for SpO$_2$ is not interpretable: the near-zero variance of the reference measurements (SD *= 0.88* percentage points post-activity) renders the difference and the mean mutually dependent by construction, an artefact discussed in Section 4.1.1.

The SpO$_2$ results illustrate why correlation alone would have been misleading. At rest, a moderate Pearson coefficient (*r = 0.35*) accompanied the smallest percentage error of any parameter (1.64%); post-activity, a weak coefficient (*r = 0.21*) accompanied the widest limits of agreement in the study. The two statistics answer different questions, and in this dataset they point in opposite directions.

**Table 3:** Bland–Altman agreement analysis

Parameter	Cond.	Bias (95% CI)	SD$_{\text {diff}}$	Lower LoA (95% CI)	Upper LoA (95% CI)
SpO$_2$ (%)	Rest	*-0.60* (*-1.56*, 0.36)	2.58	*-5.66* (*-7.33*, *-3.99*)	4.46 (2.79, 6.13)
	PA	*-0.60* (*-2.39*, 1.19)	3.82	*-8.08* (*-11.18*, *-4.99*)	6.88 (3.79, 9.97)
RR (RPM)	Rest	*-0.40* (*-0.76*, *-0.04*)	0.97	*-2.30* (*-2.93*, *-1.67*)	1.50 (0.87, 2.13)
	PA	*-0.10* (*-0.65*, 0.45)	1.17	*-2.38* (*-3.33*, *-1.44*)	2.18 (1.24, 3.13)
HR (BPM)	Rest	*-0.80* (*-1.53*, *-0.07*)	1.96	*-4.63* (*-5.90*, *-3.37*)	3.03 (1.77, 4.30)
	PA	*-1.00* (*-2.24*, 0.24)	2.66	*-6.21* (*-8.36*, *-4.05*)	4.21 (2.05, 6.36)

PA = post-activity. Rest: *n = 30*; PA: *n = 20*. Bias computed as prototype − reference; negative values indicate underestimation. LoA = 95% limits of agreement (bias $\pm 1.96\,$SD$_{\text {diff}}$). Confidence intervals computed as LoA $\pm \, t_{0.975,n-1}\sqrt{3}\,$SD$_{\text {diff}}/\sqrt{n}$ [27]

#### Sex-stratified analysis of respiratory rate

Because the optical respiratory detection mechanism transduces chest-wall displacement, its performance may depend on thoracic anatomy and breathing pattern. Respiratory-rate error was therefore stratified by sex, restricting the analysis to the rest condition (*n = 30*; 15 male, 15 female) to ensure independent observations.

Female participants showed a mean absolute percentage error of 6.67% (SD 7.92) against 2.71% (SD 4.78) in male participants—a ratio of 2.46. The difference did not reach statistical significance (Mann–Whitney *z = 1.50*, *p = 0.13*; Welch’s *t(23.0) = 1.66*, *p = 0.11*), although the effect size is moderate (Cohen’s *d = 0.61*) and with 15 participants per group the analysis is underpowered to resolve an effect of this magnitude. We therefore report this as a descriptive, hypothesis-generating observation rather than a demonstrated finding.

Bias was comparable between groups (*-0.60* vs. *-0.20* RPM), and the difference is most evident in dispersion: the 95% limits of agreement were 1.7 times wider in female participants (*-2.92* to 1.72 RPM, width 4.64) than in males (*-1.53* to 1.13 RPM, width 2.66), with correspondingly higher RMSE (1.29 vs. 0.68 RPM). The pattern is one of occasional large deviations rather than consistent underperformance: the maximum error was 27.3% in the female group against 11.8% in the male group, while most acquisitions in both groups were exact.

Should the effect prove real in a larger cohort, a plausible mechanism follows from the sensing principle. The retractable assembly transduces axial displacement of the chest wall, and two anatomical factors modulate that displacement inconsistently: a higher prevalence of diaphragmatic rather than thoracic breathing, which reduces the displacement transmitted to the sensor; and mechanical interference of breast tissue with band positioning at the intended sternal level. Both would introduce subject-dependent variability rather than a constant offset—consistent with the dispersion pattern observed. Neither is attributable to the electronics or firmware. Confirming this hypothesis requires a cohort stratified by thoracic morphology with breathing pattern classified prior to acquisition, neither of which was done here (Table 4).

**Table 4:** Sex-stratified respiratory rate performance (rest condition)

Group	*n*	MAPE (%)	RMSE (RPM)	Bias (RPM)	95% LoA (RPM)
Male	15	2.71 ± 4.78	0.68	*-0.20*	*-1.53* to 1.13
Female	15	6.67 ± 7.92	1.29	*-0.60*	*-2.92* to 1.72

MAPE = mean absolute percentage error (mean ± SD). Group comparison: Mann–Whitney *z = 1.50*, *p = 0.13*; Welch’s *t(23.0) = 1.66*, *p = 0.11*; Cohen’s *d = 0.61*. Restricted to the rest condition to preserve independence of observations

### Inertial measurement unit validation

IMU tests demonstrated discriminatory capacity of movement status. Results (Table 5) showed: sitting without movement (3%, rest status), sitting with peripheral limb movement (12%, rest status), movement sequence (35%, rest status), climbing stairs (60%, activity status), walking (64%, activity status), running (87%, activity status). The established 40% threshold effectively discriminated between rest and activity. In the tests with participants at rest, the maximum movement percentage was 4%, validating the established threshold.

**Table 5:** IMU test results in movement detection

Test type	Status	% Movement
Sitting without movement	Rest	3%
Sitting with peripheral limb movement	Rest	12%
Movement sequence	Rest	35%
Climbing stairs	Activity	60%
Walking	Activity	64%
Running	Activity	87%

### Validated measurement ranges

The ranges over which the devices were compared against reference standards were 94–100% for SpO$_2$, 7–38 RPM for respiratory rate, and 53–130 BPM for heart rate, as determined by the reference instruments. Prototype readings spanned 88–100%, 7–39 RPM, and 51–128 BPM respectively. Movement percentage ranged from 0% to 87% across the IMU protocol conditions. These are validated ranges, not operating ranges: the devices were not tested outside these bounds, and no claim is made regarding performance beyond them. This distinction matters most for SpO$_2$, where the reference values occupied a band of six percentage points at the upper physiological limit; the device has not been evaluated at any saturation below 94%.

## Discussion

### Performance of developed devices

The present study successfully developed a wearable system for capturing cardiorespiratory variables within the prespecified feasibility criterion of this study. The results demonstrate measurement errors below the ±5% criterion for all variables in this healthy-volunteer dataset, validating the feasibility of low-cost sensors for physiological monitoring in a healthy cohort. Heart rate showed the best performance (±1.94% post-physical activity), comparable to commercial devices such as the Polar OH1 [24], explained by the optimization of the MAX30105 configuration and the signal-processing algorithm.

Respiratory rate presented satisfactory results (±3.47% post-physical activity), improving over rest conditions (±4.69%), consistent with the hypothesis that greater thoracic expansion during exercise facilitates optical detection.

#### SpO$_2$ performance: the correlation–accuracy dissociation

The SpO$_2$ results contain an apparent contradiction that a reviewer of an earlier version rightly asked us to examine in depth: accuracy is good—mean absolute percentage error of 1.64% at rest and 2.98% post-activity, both well within the ±5% tolerance—yet the Pearson correlation with the reference is low (0.35 at rest, 0.21 post-activity), in stark contrast to the values above 0.96 obtained for heart rate and respiratory rate. We had previously attributed this to our prioritisation of pulse-wave quality over SpO$_2$-specific sensor configuration. That explanation is incomplete, and on reflection it cannot be the principal cause: a suboptimal sensor configuration would manifest as increased *error*, whereas our error is low. The correlation and the error are telling us different things, and the two must be analysed separately.

##### Why the correlation is low: range restriction

The Pearson coefficient is a normalised measure of covariance; it quantifies the fraction of the variance in one variable that is explained by variance in the other. It is therefore only informative when the reference variable itself varies appreciably. In our cohort of healthy young adults this condition is not met for SpO$_2$. Reference saturation spanned 94–100% at rest with a standard deviation of 1.35 percentage points, and only 96–99% post-activity with a standard deviation of 0.88—a spread comparable to the quantisation of the reference instrument itself. By contrast, reference heart rate and respiratory rate varied across 77 BPM and 31 RPM respectively, providing ample variance for correlation to be meaningful. When the true between-subject variance of the reference approaches the measurement resolution, the correlation coefficient is mathematically driven towards zero regardless of how closely the two devices agree. This is the classical phenomenon of range restriction, and its signature is unmistakable in our data: altering a single one of the twenty post-activity prototype readings by a few percentage points shifts the coefficient appreciably. A statistic this sensitive to one observation out of twenty is not characterising the device; it reflects the absence of variance to correlate. The low values are thus a statement about the physiological homogeneity of a healthy cohort, not about the instrument.

##### Why correlation is the wrong tool here

This reasoning exposes a methodological point of general relevance to device-validation studies. Correlation quantifies association, not agreement, and the two can diverge arbitrarily. As Bland and Altman established, two instruments may correlate almost perfectly while disagreeing by a large constant offset, and may agree to within measurement error while barely correlating [27]. Both situations are present in our results, and SpO$_2$ is precisely the second case: low correlation coexisting with small error. For this reason the agreement analysis reported in Section 3.5.2—not the correlation coefficient—is the appropriate basis for judging SpO$_2$ performance. At rest the Bland–Altman bias was *-0.60* percentage points (LoA *-5.66* to 4.46); post-activity the bias was likewise *-0.60* (LoA *-8.08* to 6.88). The range-restriction problem also renders the conventional Bland–Altman test for proportional bias uninterpretable for SpO$_2$, since the near-constant reference makes the difference and the mean mutually dependent by construction.

##### The genuine finding: post-exercise degradation

Separating correlation from error reveals what the correlation obscured. SpO$_2$ accuracy degraded after exercise—RMSE rose from 2.61 at rest to 3.77 post-activity, and the limits of agreement widened from a span of 10.1 to 15.0 percentage points—and this is a real effect attributable to the device rather than to the cohort. Five of twenty post-exercise acquisitions deviated by 5 percentage points or more, with a maximum underestimation of 9 points. Here the earlier explanation does apply: two mechanisms plausibly account for the degradation. First, exercise induces peripheral vasomotion and altered perfusion, changing the ratio of pulsatile to static absorption on which the ratio-of-ratios computation depends. Second, our sensor was configured to optimise pulse-wave morphology for HRV feature extraction, using an empirically tuned LED intensity threshold rather than the manufacturer’s SpO$_2$-specific configuration; this trade-off is least forgiving precisely when perfusion departs from baseline. Future firmware will implement dual-mode operation, alternating between a pulse-optimised and an oximetry-optimised configuration within a single acquisition so that neither variable is compromised for the other.

##### Boundaries of the claim

Two caveats delimit what these results support. Range restriction explains the low correlation but does not demonstrate that the device tracks saturation accurately across the clinically relevant hypoxaemic range (85–94%), which our cohort never entered: every reference value lay at or above 94%. Validation under controlled desaturation, as required by ISO 80601-2-61, remains outstanding; our device meets that standard’s accuracy root-mean-square criterion ($A_{\text {rms}} \le 4.0\%$) under both tested conditions (2.61% and 3.77%), but full conformity requires controlled desaturation testing across the 70–100% range that was not performed here, so the numerical result does not constitute conformity. We therefore make no claim of SpO$_2$ readiness for any application in which absolute saturation values inform clinical decisions. What our data establish is that, within the saturation band occupied by a healthy cohort, the device measures with an error consistent with medical-grade tolerances—a necessary, but not sufficient, precondition for the applications outlined below.

### Inertial measurement unit validation

The IMU-based movement-detection system demonstrated excellent discriminatory capacity between rest and activity states. The established 40% threshold effectively separated both conditions, with no false positives in the rest tests (maximum 4% movement). The progressive increase in movement percentage across activities (3% sitting, 35% movement sequence, 64% walking, 87% running) validates the system’s sensitivity to different intensity levels. This capability is crucial for contextualizing other physiological measurements and can be integrated with cardiovascular risk-assessment algorithms.

### System integration and data management

The mobile application successfully fulfilled its role as an integration hub for all system components. The segmentation strategy for pulse-signal transmission (4-second intervals) proved effective in managing data volume without information loss across all acquisitions, optimizing battery consumption. The cloud database integration provides a foundation for future implementation of machine-learning algorithms for heart-attack detection and prediction. Unlike studies relying exclusively on pre-existing databases [19, 20], the present system enables real-time data acquisition with patient context, potentially improving algorithm accuracy.

### Limitations and technical challenges

This work is a technical feasibility study, and its limitations should be read in that light. Its purpose was to establish measurement accuracy under controlled conditions in healthy volunteers, and its results do not extend beyond that scope.

#### Cohort composition and generalizability

Validation was restricted to 43 healthy volunteers aged 19–28 years recruited from a single university. This cohort is adequate for technical validation but severely limits generalizability, and several populations central to the system’s intended use were absent by design. No participant had a diagnosed cardiovascular condition—that is, none belonged to the population the system is ultimately intended to serve. No elderly participants wereincluded, despite the fact that pulse-wave morphology, arterial stiffness, and peripheral perfusion differ substantially in older adults and that the elderly are a primary target group for home-based cardiovascular monitoring. Although the cohort is likely to have included a range of skin phototypes and body morphologies, neither skin phototype nor body mass index was recorded, so their effect on measurement accuracy could not be analysed; given documented disparities in reflectance photoplethysmography and pulse-oximetry accuracy across skin tones, and the dependence of both the wrist PPG signal and the chest-band optical path on subcutaneous tissue thickness, this is a material omission that must be addressed before deployment. The accuracy figures reported here should therefore be regarded as an upper bound on the performance achievable in a heterogeneous clinical population, and no claim of generalizability beyond healthy young adults is made.

#### Body morphology and the respiratory sensor

Even within this cohort, morphological dependence was evident. The optical respiratory detection mechanism transduces chest-wall displacement, and its error was more variable in female participants (Section 3.5.3), attributable to a higher prevalence of diaphragmatic breathing and to mechanical interference of breast tissue with band positioning. Thoracic morphology and breathing pattern were not characterised prior to acquisition, precluding a controlled analysis of these factors. A cohort stratified by thoracic circumference, adiposity, and breast morphology is required to quantify their influence, and is a prerequisite for the screening application, which by definition must perform across the full range of adult body types.

#### Acquisition conditions

All acquisitions were performed over 60 s with the participant supine and motionless, including in the post-activity group, which was measured after exercise rather than during it. Performance under ambulatory conditions—where motion artefact is the dominant error source for both PPG and optical respiratory sensing—was not assessed. The demonstrated accuracy therefore applies to controlled, stationary acquisitions and does not extend to continuous free-living monitoring.

#### SpO$_2$ saturation range

Reference saturation never fell below 94% (Section 4.1.1). The device has not been evaluated across the hypoxaemic range in which SpO$_2$ carries clinical consequence, and validation under controlled desaturation per ISO 80601-2-61 remains outstanding.

#### Methodological limitations

The activity-classification threshold was derived empirically rather than by formal optimisation. Peak-detection parameters were likewise fixed empirically rather than optimised against annotated reference data, although identical parameters were applied to the prototype and reference signals (Section 2.7). The sex-stratified comparison was underpowered (Section 3.5.3).

#### Engineering validation not performed

Long-term signal stability, battery life under continuous use, electromagnetic compatibility, ingress protection, biocompatibility of the enclosure materials, and durability under daily wear were not evaluated. The three low-battery incidents encountered during data collection, which produced physiologically implausible readings and required reacquisition, underscore that supply-state monitoring and a signal-quality index are prerequisites for unsupervised use: a low-battery artefact was not distinguishable from a genuine desaturation on the basis of the reported value alone.

### Comparison with existing technologies

Compared to ECG-based devices, the present system offers advantages in user comfort by eliminating adhesive electrodes [9], at the cost of losing cardiac electrical information. The photoplethysmography approach positions the proposed system between consumer-grade fitness trackers and clinical monitoring devices. Unlike the Apple Watch or Garmin Smartwatch [21, 22], which do not provide raw PPG signals, the proposed system provides complete waveforms for advanced processing. The integration of respiratory-rate measurement and system architecture specifically designed for heart-attack detection represents a unique contribution.

In terms of heart-rate accuracy, the proposed system achieved ±1.94% error (*r = 0.99*), comparable to leading wrist-worn PPG devices such as the Polar OH1+ [24]. For respiratory rate, our system attained ±3.47% error, outperforming several impedance-based wearable systems reported in the literature [26], which typically achieve RMSE values of 1.5–3.5 breaths/min under ambulatory conditions; our RMSE of 1.14 breaths/min post-activity is lower, attributable to the direct mechanical respiratory-detection approach, which is less susceptible to motion-induced degradation than PPG-derived or accelerometry-based estimation. With respect to SpO$_2$, our device achieved ±2.98% post-activity, consistent with the performance range reported for consumer-grade optical sensors (±2–5%) but above the ±2% threshold recommended for clinical-grade pulse oximeters. These comparisons confirm that the proposed low-cost system achieves performance within clinically acceptable benchmarks for non-critical monitoring, while acknowledging areas for future improvement, particularly for SpO$_2$.

### Clinical implications and future directions

The measurement accuracy obtained supports the technical feasibility of the system for the applications outlined in Section 1. The integration of heart rate, HRV, SpO$_2$, respiratory rate, movement status, and patient context provides a physiological profile that could support cardiovascular risk stratification [8]. Translating this technical feasibility into clinical utility, however, requires a staged pathway, and it is worth stating explicitly which stages are complete and which are not.

#### Stage 1—technical validation (this study)

Accuracy characterisation against clinical-grade reference equipment in healthy volunteers under controlled rest and post-exercise conditions. Complete.

#### Stage 2—data collection in the target population (in progress)

A study using the system described here is under way in collaboration with a consultant cardiologist at Clínica Valle de Pubenza (Popayán, Cauca, Colombia). To date 50 high-risk older adults have been assessed, each contributing physiological signals acquired with the present system alongside clinical data, demographic information, and a specialist-assigned short-term myocardial-infarction risk level established from concurrent electrocardiography and biomarker testing. Recruitment is ongoing.

#### Stage 3—model development (in progress; preliminary)

A preliminary risk-estimation model has been trained on this cohort. Its performance does not yet reach the range required for any clinical application, which we attribute primarily to the size of the labelled dataset relative to the dimensionality of the feature space; cohort expansion is the current priority. We report this preliminary negative result deliberately, since the contribution of the present paper is the validated acquisition platform, and overstating the maturity of the downstream algorithm would misrepresent the state of the work.

#### Stage 4—prospective clinical evaluation (not started)

Evaluation of the resulting model against clinical endpoints, with pre-specified sensitivity and specificity targets appropriate to a screening instrument, must precede any consideration of deployment or regulatory submission.

The single most important direction for future work is validation in the target clinical populations. The accuracy established here in healthy young adults is a necessary precondition but not evidence of clinical performance, and the next stage must recruit deliberately for the diversity absent from the present cohort: older adults, patients with diagnosed cardiovascular disease, and participants spanning the range of skin phototypes, body-mass indices, and thoracic morphologies present in the general adult population. Anthropometric variables and skin phototype, not recorded here, will be captured prospectively so that their influence on measurement accuracy can be quantified rather than merely acknowledged. Only after such validation would performance claims for screening or clinical monitoring be warranted. Subsequent work will report the feature-extraction pipeline applied to the stored waveforms and its performance in risk-estimation models; hardware iterations targeting pulse-waveform morphology will adopt a higher acquisition rate, which the existing firmware already supports. The intended applications are twofold: rapid, low-burden cardiovascular screening of adults in settings where access to specialist cardiology is limited, with high-risk individuals referred for conventional assessment; and home-based longitudinal monitoring of patients already diagnosed as high cardiovascular risk. System cost is material to both applications, since mass screening is only meaningful if the instrument is affordable at the scale of the population that needs it. The components of both devices total $82.88 USD at single-unit international distributor prices (Appendix A), excluding assembly labour and printed-circuit-board fabrication. This figure is a conservative upper bound: it reflects the most expensive procurement route available, whereas the prototypes were built from regionally sourced components, and unit cost would fall substantially at production volume. Even so, it remains between one and two orders of magnitude below the clinical instruments against which the system was validated, and below consumer devices offering fewer physiological variables and no access to raw waveforms [21, 22].

Concurrent electroencephalography would additionally permit brain–heart coupling to be examined, since cardiorespiratory efficiency modulates cerebral oxygenation through cardiac output and heart-rate variability has been related to electroencephalographic activity in cardiovascular contexts [29]; established connectivity and graph-theoretic network metrics [28] could then establish whether autonomic dysregulation preceding myocardial events is detectable in this way. This would require a controlled laboratory setting rather than the ambulatory use targeted here, though it operates on the inter-beat interval series the system already stores.

### Contributions and significance

This work contributes to wearable health monitoring by proposing a low-cost, integrated sensor system that achieves clinically acceptable measurements for multiple cardiorespiratory variables in a healthy cohort. The novel optical respiratory-detection mechanism represents a simple yet effective alternative to traditional sensors. The provision of raw physiological signals, combined with patient context and cloud storage, creates a framework suitable for advanced algorithms for heart-attack detection and prediction, addressing a critical limitation in previous studies [19, 20]. The integration of movement detection with cardiorespiratory monitoring enables contextualized interpretation of physiological changes, potentially reducing false alarms.

## Conclusion

This work introduces a low-cost, integrated wearable system capable of acquiring multiple cardiorespiratory variables with accuracy meeting the study’s prespecified feasibility criterion in healthy volunteers. The combination of photoplethysmographic pulse sensing, optical respiratory detection, inertial movement assessment, mobile application, and cloud database yields a comprehensive architecture tailored to cardiovascular risk assessment. By providing access to raw physiological signals together with contextual information, the platform bridges the gap between consumer-grade fitness trackers and specialized medical monitoring systems and offers a practical basis for implementing and validating advanced machine-learning algorithms for early detection and prediction of myocardial infarction in future clinical studies.

As a feasibility study in healthy young volunteers, this work establishes technical performance but not clinical validity; validation in elderly and cardiovascular patient populations, across the full range of skin phototypes and body morphologies, is the necessary next step. The system demonstrated clinically acceptable performance for heart rate (±1.94%, *r = 0.99*) and respiratory rate (±3.47%, *r = 0.99*) monitoring, while SpO$_2$ measurement requires further optimization and validation across the hypoxaemic range before deployment in applications where absolute saturation values drive clinical decisions. The validated platform establishes a foundation for developing context-aware cardiovascular risk-assessment algorithms that could contribute to reducing mortality from acute myocardial infarction through early detection.

## Supplementary Information

Below is the link to the electronic supplementary material.

Supplementary file 1 (xlsx 21 KB)

## Figures and Tables

**Algorithm 1:**
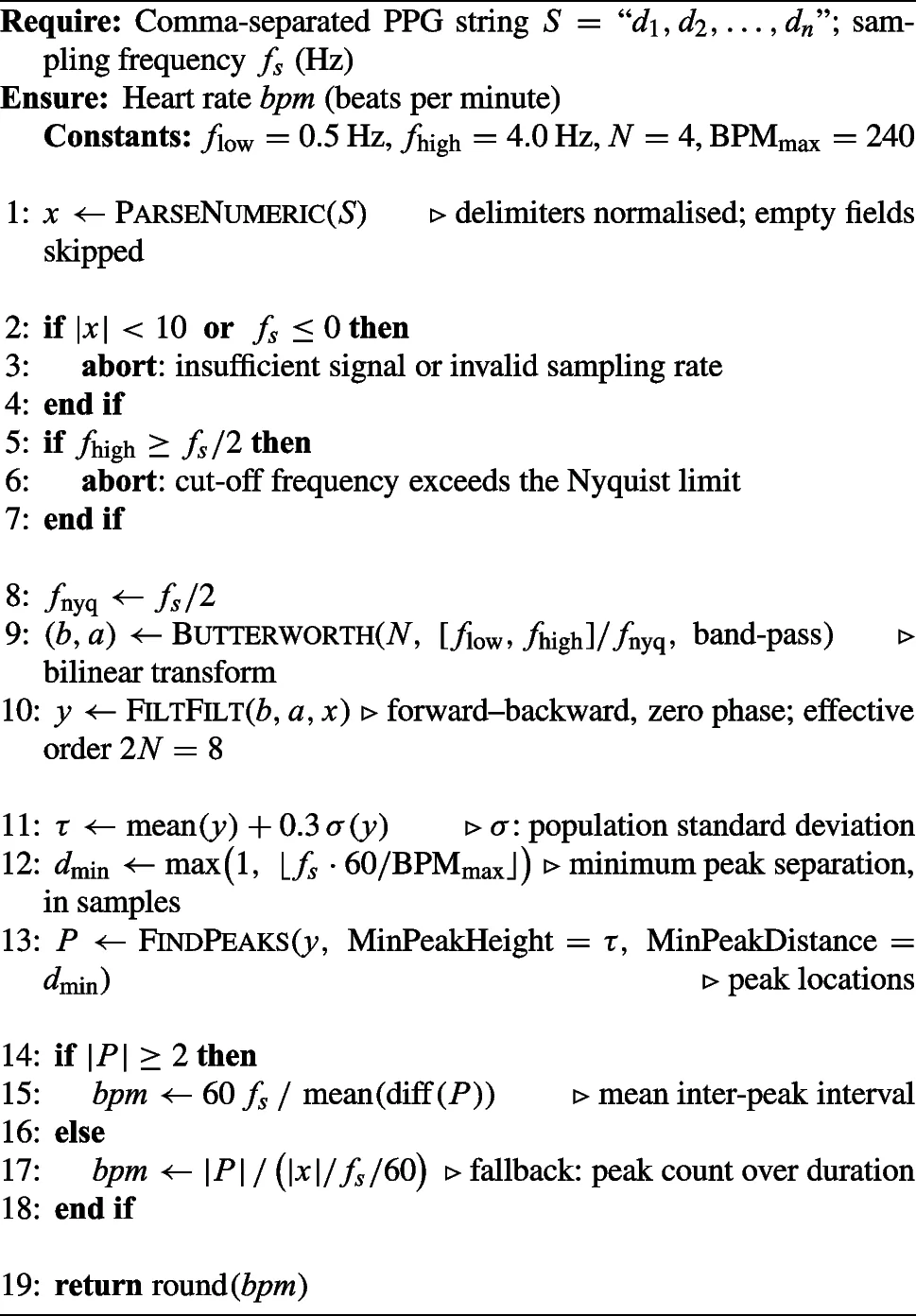
Heart-rate estimation from the photoplethysmographic signal.

## Data Availability

The complete raw physiological dataset supporting this study is provided as Supplementary Material to permit independent verification. Additional processed data are available from the corresponding author upon reasonable request, subject to ethical restrictions on individual participant data.

## Appendix A: Bill of materials

**Table 6:** Component costs for both devices

Module	Component (quoted part)	Cost (USD)
Wristband	Photoplethysmographic sensor (Pimoroni PIM438)^a^	23.28
	Microcontroller (Seeed Studio 113991054)^a,b^	5.25
	Watch straps (M5Stack A128-B)^a^	7.50
Chest band	Inertial measurement unit (TDK ICM-42686-P)^a^	6.24
	Microcontroller (Seeed Studio 113991054)^a,b^	5.25
	Red LED, 3 mm (ams-OSRAM LS E67F)	0.34
	Photoresistor (Soldered 101110)	0.54
	Resistors (KOA Speer PCF1-2C181K)^a^	1.24
	Strap and fastening (commercial chest strap)^a,c^	7.58
Both	Rechargeable batteries, *2× * Li-Po 3.7 V 115 mAh	
	(Renata ICP501421PS)^a^	23.18
	3D-printed enclosures^d^	2.48
Total		82.88

Prices retrieved on 23 July 2026 from a single international distributor (Mouser Electronics) at single-unit retail rates, except where indicated. The total is therefore a conservative upper bound rather than the amount actually spent: the prototypes were assembled from regionally sourced components, and unit cost would decrease substantially at production volume.

^a^The exact component used in the prototype was not listed by this distributor at the time of quotation; the closest functionally equivalent part is quoted instead. These entries establish an order-of-magnitude cost rather than an exact reproduction of the prototype bill of materials.

^b^Two microcontroller boards were purchased for $10.50; the cost is divided evenly between the two devices.

^c^Quoted from a general-purpose retailer rather than an electronics distributor, as no equivalent part number was available.

^d^Quoted from an online 3D-printing service (JLC3DP) for the seven enclosure parts of both devices, totalling 0.19 kg in SLA resin.

Assembly labour, printed-circuit-board fabrication, soldering consumables, and amortisation of laboratory equipment are excluded throughout.

## Appendix B: Heart-rate estimation algorithm

Algorithm 1 details the procedure described in Section 2.7, by which heart rate is obtained from the stored photoplethysmographic waveform.

Implemented in MATLAB R2024b using the Signal Processing Toolbox (butter, filtfilt, findpeaks). The routine was invoked with $f_s = 10$ Hz, the measured effective acquisition rate of the wristband device. The band-pass limits of 0.5 and 4.0 Hz correspond to a physiological range of 30 to 240 beats per minute, and the same 240 beats-per-minute ceiling defines the minimum admissible separation between consecutive peaks.
